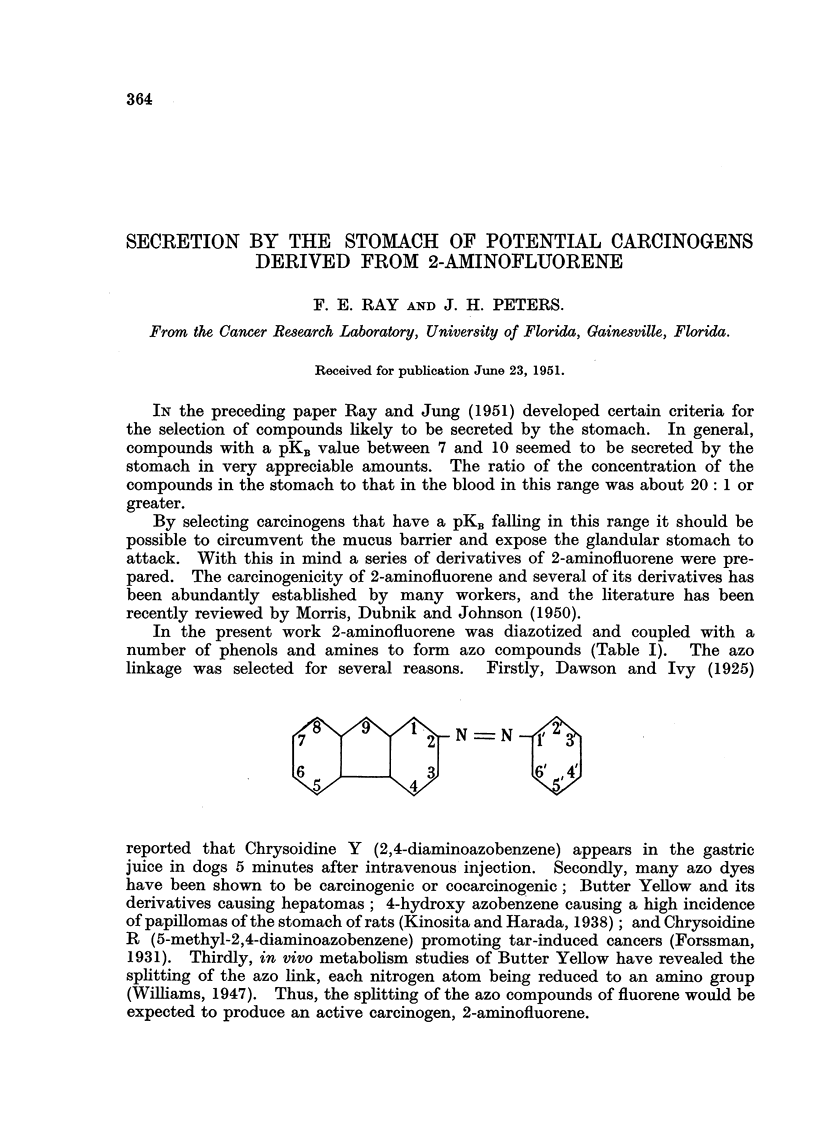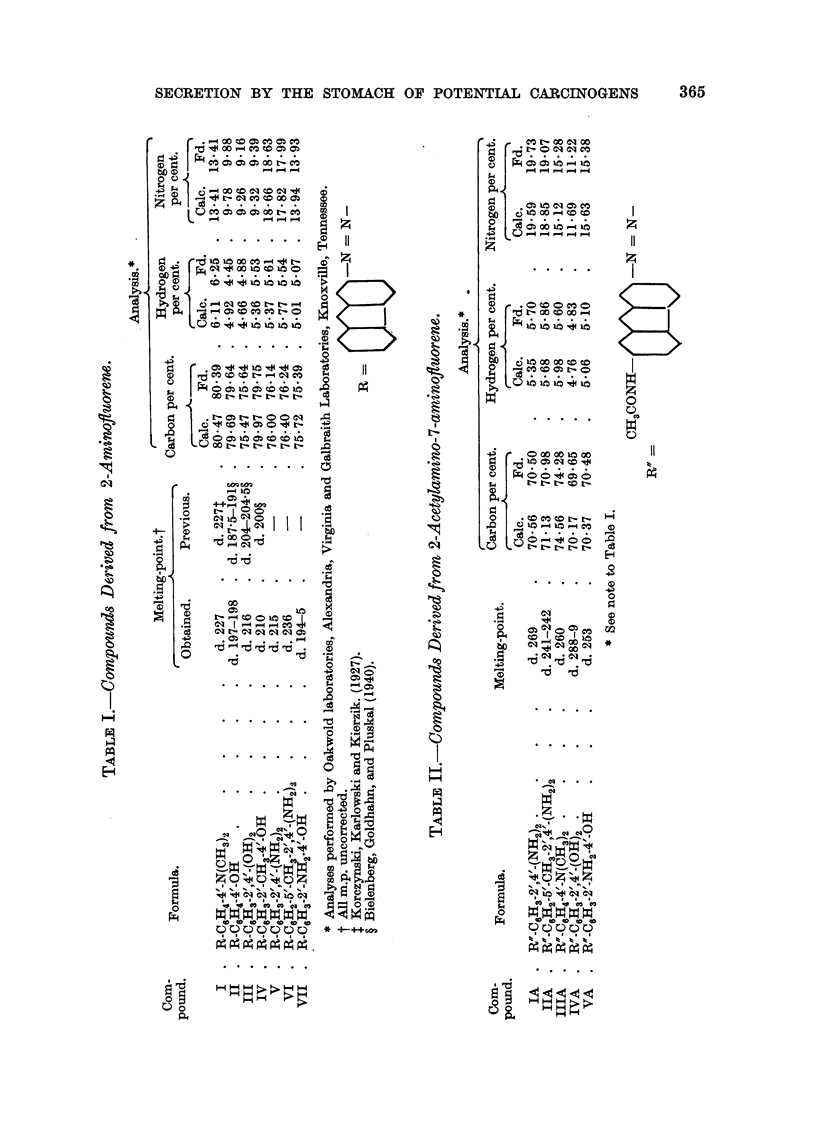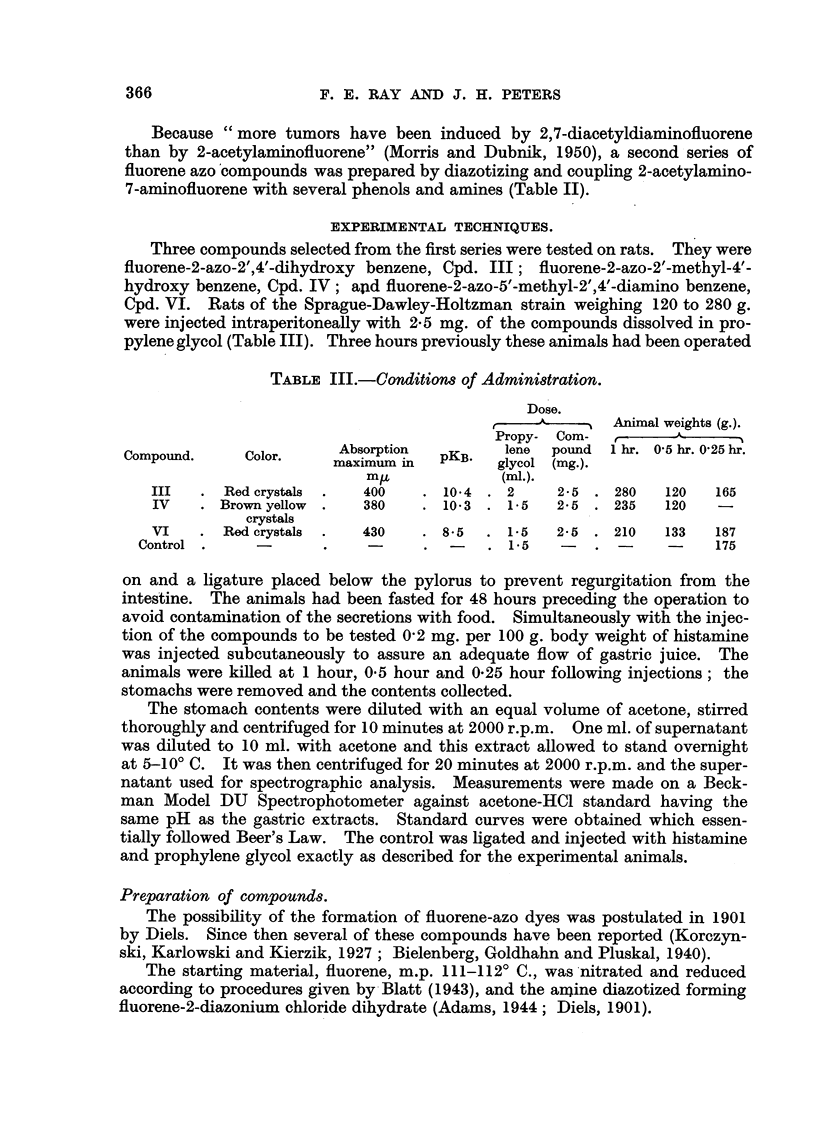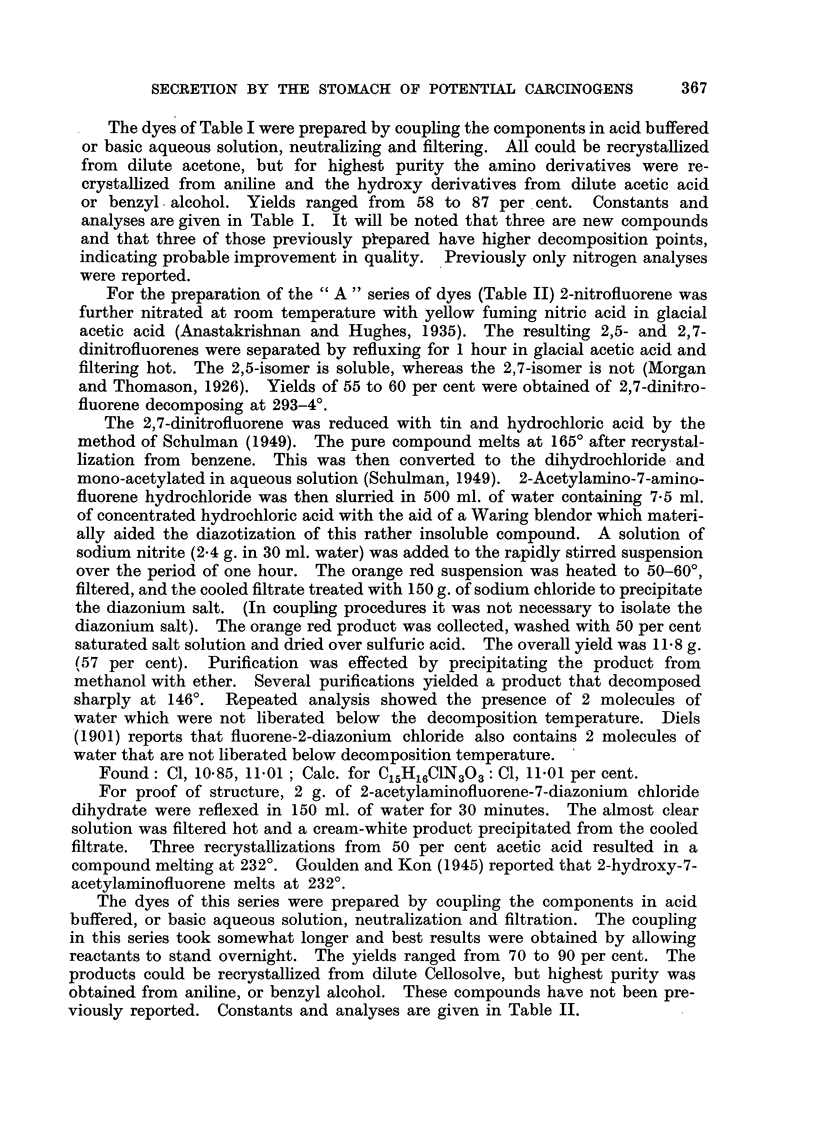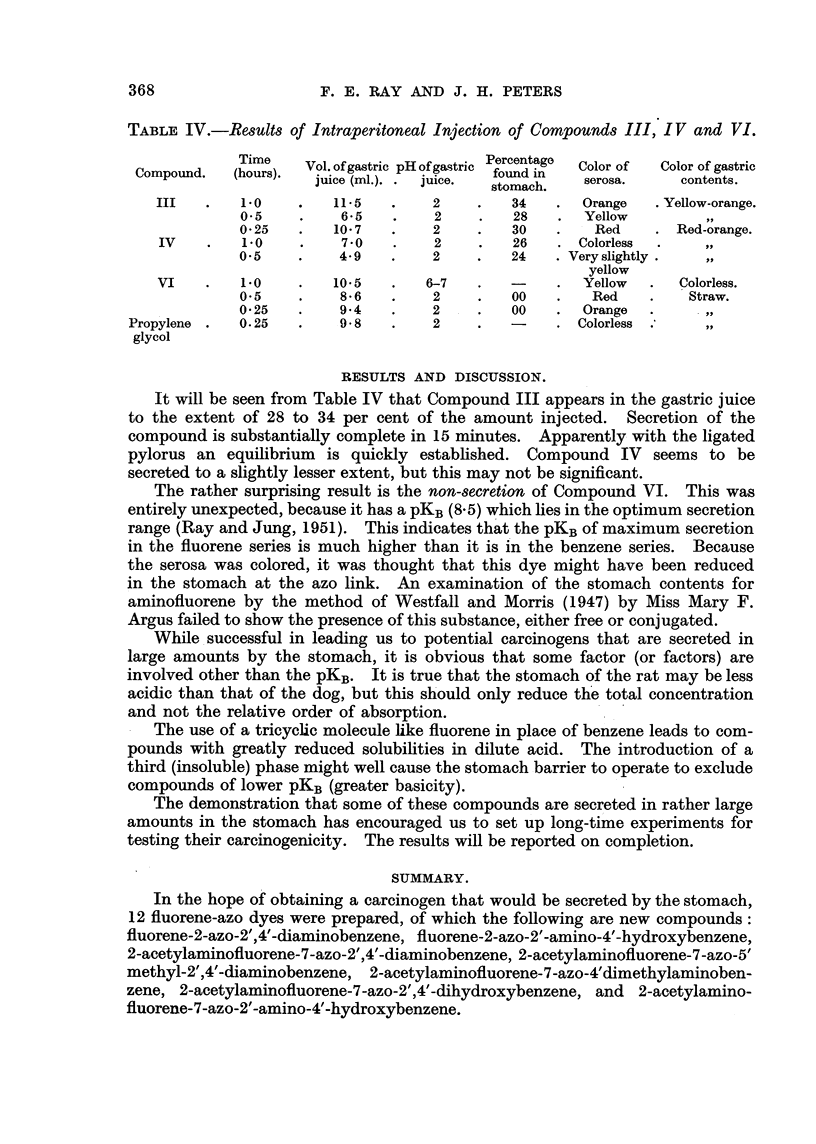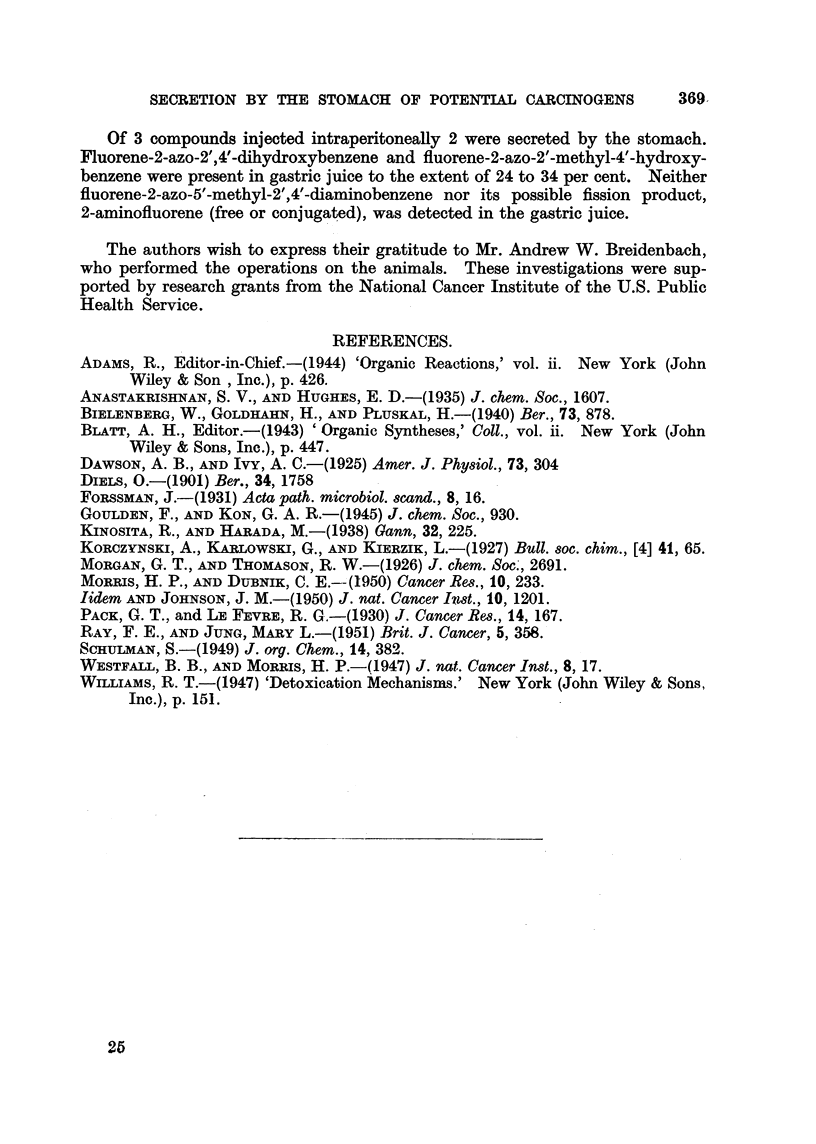# Secretion by the Stomach of Potential Carcinogens Derived from 2-Aminofluorene

**DOI:** 10.1038/bjc.1951.39

**Published:** 1951-09

**Authors:** F. E. Ray, J. H. Peters


### SECRETION BY THE STOMACH OF POTENTIAL CARCINOGENS DERIVED FROM 2-AMINOFLUORENE

F. E. RAY AND J. H. PETERS.

*From the Cancer Research Laboratory, University of Florida, Gainesville, Florida.*

Received for publication June 23, 1951.

IN the preceding paper Ray and Jung (1951) developed certain criteria for the selection of compounds likely to be secreted by the stomach. In general, compounds with a $pK_B$ value between 7 and 10 seemed to be secreted by the stomach in very appreciable amounts. The ratio of the concentration of the compounds in the stomach to that in the blood in this range was about 20 : 1 or greater.

By selecting carcinogens that have a $pK_B$ falling in this range it should be possible to circumvent the mucus barrier and expose the glandular stomach to attack. With this in mind a series of derivatives of 2-aminofluorene were prepared. The carcinogenicity of 2-aminofluorene and several of its derivatives has been abundantly established by many workers, and the literature has been recently reviewed by Morris, Dubnik and Johnson (1950).

In the present work 2-aminofluorene was diazotized and coupled with a number of phenols and amines to form azo compounds (Table I). The azo linkage was selected for several reasons. Firstly, Dawson and Ivy (1925)

reported that Chrysoidine Y (2,4-diaminoazobenzene) appears in the gastric juice in dogs 5 minutes after intravenous injection. Secondly, many azo dyes have been shown to be carcinogenic or cocarcinogenic ; Butter Yellow and its derivatives causing hepatomas ; 4-hydroxy azobenzene causing a high incidence of papillomas of the stomach of rats (Kinosita and Harada, 1938) ; and Chrysoidine R (5-methyl-2,4-diaminoazobenzene) promoting tar-induced cancers (Forssman, 1931). Thirdly, *in vivo* metabolism studies of Butter Yellow have revealed the splitting of the azo link, each nitrogen atom being reduced to an amino group (Williams, 1947). Thus, the splitting of the azo compounds of fluorene would be expected to produce an active carcinogen, 2-aminofluorene.

TABLE I.—*Compounds Derived from 2-Aminofluorene.*

| Compound. | Formula. | Melting-point.† Obtained. | Melting-point.† Previous. | Analysis.* Carbon per cent. Calc. | Carbon per cent. Fd. | Hydrogen per cent. Calc. | Hydrogen per cent. Fd. | Nitrogen per cent. Calc. | Nitrogen per cent. Fd. |
|---|---|---|---|---|---|---|---|---|---|
| I | $R\text{-}C_6H_4\text{-}4'\text{-}N(CH_3)_2$ | d. 227 | d. 227‡ | 80·47 | 80·39 | 6·11 | 6·25 | 13·41 | 13·41 |
| II | $R\text{-}C_6H_4\text{-}4'\text{-}OH$ | d. 197–198 | d. 187·5–191§ | 79·69 | 79·64 | 4·92 | 4·45 | 9·78 | 9·88 |
| III | $R\text{-}C_6H_3\text{-}2',4'\text{-}(OH)_2$ | d. 216 | d. 204–204·5§ | 75·47 | 75·64 | 4·66 | 4·88 | 9·26 | 9·16 |
| IV | $R\text{-}C_6H_3\text{-}2'\text{-}CH_3\text{-}4'\text{-}OH$ | d. 210 | d. 200§ | 79·97 | 79·75 | 5·36 | 5·53 | 9·32 | 9·39 |
| V | $R\text{-}C_6H_3\text{-}2',4'\text{-}(NH_2)_2$ | d. 215 | — | 76·00 | 76·14 | 5·37 | 5·61 | 18·66 | 18·63 |
| VI | $R\text{-}C_6H_2\text{-}5'\text{-}CH_3\text{-}2',4'\text{-}(NH_2)_2$ | d. 236 | — | 76·40 | 76·24 | 5·77 | 5·54 | 17·82 | 17·99 |
| VII | $R\text{-}C_6H_3\text{-}2'\text{-}NH_2\text{-}4'\text{-}OH$ | d. 194–5 | — | 75·72 | 75·39 | 5·01 | 5·07 | 13·94 | 13·93 |

* Analyses performed by Oakwold laboratories, Alexandria, Virginia and Galbraith Laboratories, Knoxville, Tennessee.

† All m.p. uncorrected.

‡ Korczynski, Karlowski and Kierzik. (1927).

§ Bielenberg, Goldhahn, and Pluskal (1940).

R =  —N = N—

TABLE II.—*Compounds Derived from 2-Acetylamino-7-aminofluorene.*

| Compound. | Formula. | Melting-point. | Analysis.* Carbon per cent. Calc. | Carbon per cent. Fd. | Hydrogen per cent. Calc. | Hydrogen per cent. Fd. | Nitrogen per cent. Calc. | Nitrogen per cent. Fd. |
|---|---|---|---|---|---|---|---|---|
| IA | $R''\text{-}C_6H_3\text{-}2',4'\text{-}(NH_2)_2$ | d. 269 | 70·56 | 70·50 | 5·35 | 5·70 | 19·59 | 19·73 |
| IIA | $R''\text{-}C_6H_2\text{-}5'\text{-}CH_3\text{-}2',4'\text{-}(NH_2)_2$ | d. 241–242 | 71·13 | 70·98 | 5·68 | 5·86 | 18·85 | 19·07 |
| IIIA | $R''\text{-}C_6H_4\text{-}4'\text{-}N(CH_3)_2$ | d. 260 | 74·56 | 74·28 | 5·98 | 5·60 | 15·12 | 15·28 |
| IVA | $R''\text{-}C_6H_3\text{-}2',4'\text{-}(OH)_2$ | d. 288–9 | 70·17 | 69·65 | 4·76 | 4·83 | 11·69 | 11·22 |
| VA | $R''\text{-}C_6H_3\text{-}2'\text{-}NH_2\text{-}4'\text{-}OH$ | d. 253 | 70·37 | 70·48 | 5·06 | 5·10 | 15·63 | 15·38 |

* See note to Table I.

R″ = $CH_3CONH$—  —N = N—

Because "more tumors have been induced by 2,7-diacetyldiaminofluorene than by 2-acetylaminofluorene" (Morris and Dubnik, 1950), a second series of fluorene azo compounds was prepared by diazotizing and coupling 2-acetylamino-7-aminofluorene with several phenols and amines (Table II).

### EXPERIMENTAL TECHNIQUES.

Three compounds selected from the first series were tested on rats. They were fluorene-2-azo-2′,4′-dihydroxy benzene, Cpd. III; fluorene-2-azo-2′-methyl-4′-hydroxy benzene, Cpd. IV; and fluorene-2-azo-5′-methyl-2′,4′-diamino benzene, Cpd. VI. Rats of the Sprague-Dawley-Holtzman strain weighing 120 to 280 g. were injected intraperitoneally with 2·5 mg. of the compounds dissolved in propylene glycol (Table III). Three hours previously these animals had been operated on and a ligature placed below the pylorus to prevent regurgitation from the intestine. The animals had been fasted for 48 hours preceding the operation to avoid contamination of the secretions with food. Simultaneously with the injection of the compounds to be tested 0·2 mg. per 100 g. body weight of histamine was injected subcutaneously to assure an adequate flow of gastric juice. The animals were killed at 1 hour, 0·5 hour and 0·25 hour following injections; the stomachs were removed and the contents collected.

TABLE III.—*Conditions of Administration.*

| Compound. | Color. | Absorption maximum in mμ | $pK_B$. | Dose. Propylene glycol (ml.). | Compound (mg.). | Animal weights (g.). 1 hr. | 0·5 hr. | 0·25 hr. |
|---|---|---|---|---|---|---|---|---|
| III | Red crystals | 400 | 10·4 | 2 | 2·5 | 280 | 120 | 165 |
| IV | Brown yellow crystals | 380 | 10·3 | 1·5 | 2·5 | 235 | 120 | — |
| VI | Red crystals | 430 | 8·5 | 1·5 | 2·5 | 210 | 133 | 187 |
| Control | — | — | — | 1·5 | — | — | — | 175 |

The stomach contents were diluted with an equal volume of acetone, stirred thoroughly and centrifuged for 10 minutes at 2000 r.p.m. One ml. of supernatant was diluted to 10 ml. with acetone and this extract allowed to stand overnight at 5–10° C. It was then centrifuged for 20 minutes at 2000 r.p.m. and the supernatant used for spectrographic analysis. Measurements were made on a Beckman Model DU Spectrophotometer against acetone-HCl standard having the same pH as the gastric extracts. Standard curves were obtained which essentially followed Beer's Law. The control was ligated and injected with histamine and prophylene glycol exactly as described for the experimental animals.

### *Preparation of compounds.*

The possibility of the formation of fluorene-azo dyes was postulated in 1901 by Diels. Since then several of these compounds have been reported (Korczynski, Karlowski and Kierzik, 1927; Bielenberg, Goldhahn and Pluskal, 1940).

The starting material, fluorene, m.p. 111–112° C., was nitrated and reduced according to procedures given by Blatt (1943), and the amine diazotized forming fluorene-2-diazonium chloride dihydrate (Adams, 1944; Diels, 1901).

The dyes of Table I were prepared by coupling the components in acid buffered or basic aqueous solution, neutralizing and filtering. All could be recrystallized from dilute acetone, but for highest purity the amino derivatives were recrystallized from aniline and the hydroxy derivatives from dilute acetic acid or benzyl alcohol. Yields ranged from 58 to 87 per cent. Constants and analyses are given in Table I. It will be noted that three are new compounds and that three of those previously prepared have higher decomposition points, indicating probable improvement in quality. Previously only nitrogen analyses were reported.

For the preparation of the " A " series of dyes (Table II) 2-nitrofluorene was further nitrated at room temperature with yellow fuming nitric acid in glacial acetic acid (Anastakrishnan and Hughes, 1935). The resulting 2,5- and 2,7-dinitrofluorenes were separated by refluxing for 1 hour in glacial acetic acid and filtering hot. The 2,5-isomer is soluble, whereas the 2,7-isomer is not (Morgan and Thomason, 1926). Yields of 55 to 60 per cent were obtained of 2,7-dinitrofluorene decomposing at 293–4°.

The 2,7-dinitrofluorene was reduced with tin and hydrochloric acid by the method of Schulman (1949). The pure compound melts at 165° after recrystallization from benzene. This was then converted to the dihydrochloride and mono-acetylated in aqueous solution (Schulman, 1949). 2-Acetylamino-7-aminofluorene hydrochloride was then slurried in 500 ml. of water containing 7·5 ml. of concentrated hydrochloric acid with the aid of a Waring blendor which materially aided the diazotization of this rather insoluble compound. A solution of sodium nitrite (2·4 g. in 30 ml. water) was added to the rapidly stirred suspension over the period of one hour. The orange red suspension was heated to 50–60°, filtered, and the cooled filtrate treated with 150 g. of sodium chloride to precipitate the diazonium salt. (In coupling procedures it was not necessary to isolate the diazonium salt). The orange red product was collected, washed with 50 per cent saturated salt solution and dried over sulfuric acid. The overall yield was 11·8 g. (57 per cent). Purification was effected by precipitating the product from methanol with ether. Several purifications yielded a product that decomposed sharply at 146°. Repeated analysis showed the presence of 2 molecules of water which were not liberated below the decomposition temperature. Diels (1901) reports that fluorene-2-diazonium chloride also contains 2 molecules of water that are not liberated below decomposition temperature.

Found : Cl, 10·85, 11·01 ; Calc. for $C_{15}H_{16}ClN_3O_3$ : Cl, 11·01 per cent.

For proof of structure, 2 g. of 2-acetylaminofluorene-7-diazonium chloride dihydrate were reflexed in 150 ml. of water for 30 minutes. The almost clear solution was filtered hot and a cream-white product precipitated from the cooled filtrate. Three recrystallizations from 50 per cent acetic acid resulted in a compound melting at 232°. Goulden and Kon (1945) reported that 2-hydroxy-7-acetylaminofluorene melts at 232°.

The dyes of this series were prepared by coupling the components in acid buffered, or basic aqueous solution, neutralization and filtration. The coupling in this series took somewhat longer and best results were obtained by allowing reactants to stand overnight. The yields ranged from 70 to 90 per cent. The products could be recrystallized from dilute Cellosolve, but highest purity was obtained from aniline, or benzyl alcohol. These compounds have not been previously reported. Constants and analyses are given in Table II.

TABLE IV.—*Results of Intraperitoneal Injection of Compounds III, IV and VI.*

| Compound. | Time (hours). | Vol. of gastric juice (ml.). | pH of gastric juice. | Percentage found in stomach. | Color of serosa. | Color of gastric contents. |
|---|---|---|---|---|---|---|
| III | 1·0 | 11·5 | 2 | 34 | Orange | Yellow-orange. |
| | 0·5 | 6·5 | 2 | 28 | Yellow | ,, |
| | 0·25 | 10·7 | 2 | 30 | Red | Red-orange. |
| IV | 1·0 | 7·0 | 2 | 26 | Colorless | ,, |
| | 0·5 | 4·9 | 2 | 24 | Very slightly yellow | ,, |
| VI | 1·0 | 10·5 | 6–7 | — | Yellow | Colorless. |
| | 0·5 | 8·6 | 2 | 00 | Red | Straw. |
| | 0·25 | 9·4 | 2 | 00 | Orange | ,, |
| Propylene glycol | 0.25 | 9·8 | 2 | — | Colorless | ,, |

### RESULTS AND DISCUSSION.

It will be seen from Table IV that Compound III appears in the gastric juice to the extent of 28 to 34 per cent of the amount injected. Secretion of the compound is substantially complete in 15 minutes. Apparently with the ligated pylorus an equilibrium is quickly established. Compound IV seems to be secreted to a slightly lesser extent, but this may not be significant.

The rather surprising result is the *non-secretion* of Compound VI. This was entirely unexpected, because it has a $pK_B$ (8·5) which lies in the optimum secretion range (Ray and Jung, 1951). This indicates that the $pK_B$ of maximum secretion in the fluorene series is much higher than it is in the benzene series. Because the serosa was colored, it was thought that this dye might have been reduced in the stomach at the azo link. An examination of the stomach contents for aminofluorene by the method of Westfall and Morris (1947) by Miss Mary F. Argus failed to show the presence of this substance, either free or conjugated.

While successful in leading us to potential carcinogens that are secreted in large amounts by the stomach, it is obvious that some factor (or factors) are involved other than the $pK_B$. It is true that the stomach of the rat may be less acidic than that of the dog, but this should only reduce the total concentration and not the relative order of absorption.

The use of a tricyclic molecule like fluorene in place of benzene leads to compounds with greatly reduced solubilities in dilute acid. The introduction of a third (insoluble) phase might well cause the stomach barrier to operate to exclude compounds of lower $pK_B$ (greater basicity).

The demonstration that some of these compounds are secreted in rather large amounts in the stomach has encouraged us to set up long-time experiments for testing their carcinogenicity. The results will be reported on completion.

### SUMMARY.

In the hope of obtaining a carcinogen that would be secreted by the stomach, 12 fluorene-azo dyes were prepared, of which the following are new compounds : fluorene-2-azo-2′,4′-diaminobenzene, fluorene-2-azo-2′-amino-4′-hydroxybenzene, 2-acetylaminofluorene-7-azo-2′,4′-diaminobenzene, 2-acetylaminofluorene-7-azo-5′ methyl-2′,4′-diaminobenzene, 2-acetylaminofluorene-7-azo-4′dimethylaminobenzene, 2-acetylaminofluorene-7-azo-2′,4′-dihydroxybenzene, and 2-acetylaminofluorene-7-azo-2′-amino-4′-hydroxybenzene.

Of 3 compounds injected intraperitoneally 2 were secreted by the stomach. Fluorene-2-azo-2′,4′-dihydroxybenzene and fluorene-2-azo-2′-methyl-4′-hydroxybenzene were present in gastric juice to the extent of 24 to 34 per cent. Neither fluorene-2-azo-5′-methyl-2′,4′-diaminobenzene nor its possible fission product, 2-aminofluorene (free or conjugated), was detected in the gastric juice.

The authors wish to express their gratitude to Mr. Andrew W. Breidenbach, who performed the operations on the animals. These investigations were supported by research grants from the National Cancer Institute of the U.S. Public Health Service.

REFERENCES.

Adams, R., Editor-in-Chief.—(1944) 'Organic Reactions,' vol. ii. New York (John Wiley & Son , Inc.), p. 426.
Anastakrishnan, S. V., and Hughes, E. D.—(1935) *J. chem. Soc.*, 1607.
Bielenberg, W., Goldhahn, H., and Pluskal, H.—(1940) *Ber.*, **73**, 878.
Blatt, A. H., Editor.—(1943) 'Organic Syntheses,' *Coll.*, vol. ii. New York (John Wiley & Sons, Inc.), p. 447.
Dawson, A. B., and Ivy, A. C.—(1925) *Amer. J. Physiol.*, **73**, 304
Diels, O.—(1901) *Ber.*, **34**, 1758
Forssman, J.—(1931) *Acta path. microbiol. scand.*, **8**, 16.
Goulden, F., and Kon, G. A. R.—(1945) *J. chem. Soc.*, 930.
Kinosita, R., and Harada, M.—(1938) *Gann*, **32**, 225.
Korczynski, A., Karlowski, G., and Kierzik, L.—(1927) *Bull. soc. chim.*, [4] **41**, 65.
Morgan, G. T., and Thomason, R. W.—(1926) *J. chem. Soc.*, 2691.
Morris, H. P., and Dubnik, C. E.—(1950) *Cancer Res.*, **10**, 233.
*Iidem* and Johnson, J. M.—(1950) *J. nat. Cancer Inst.*, **10**, 1201.
Pack, G. T., and Le Fevre, R. G.—(1930) *J. Cancer Res.*, **14**, 167.
Ray, F. E., and Jung, Mary L.—(1951) *Brit. J. Cancer*, **5**, 358.
Schulman, S.—(1949) *J. org. Chem.*, **14**, 382.
Westfall, B. B., and Morris, H. P.—(1947) *J. nat. Cancer Inst.*, **8**, 17.
Williams, R. T.—(1947) 'Detoxication Mechanisms.' New York (John Wiley & Sons, Inc.), p. 151.